# Clinical and pathological characteristics of giant cell angioblastoma: a case report

**DOI:** 10.1186/1746-1596-7-113

**Published:** 2012-08-29

**Authors:** Rong-Jun Mao, Zhi-Ming Jiang, Hui-Zhen Zhang, Xiong-Zeng Zhu, Qing-Ling Zhang

**Affiliations:** 1 Department of Pathology, Foshan Hospital of Traditional Chinese Medicine, Guangzhou University of Chinese Medicine, Foshan, People’s Republic of China; 2 Department of Pathology, Shanghai Sixth People’s Hospital Affiliated to Shanghai Jiaotong University, Shanghai, People’s Republic of China; 3 Department of Pathology, Cancer Hospital, Fudan University, Shanghai, People’s Republic of China; 4 Department of Pathology, Nanfang Hospital, Southern Medical University, Guangzhou, People’s Republic of China; 5 Department of Pathology, College of Basic Medicine, Southern Medical University, Guangzhou, People’s Republic of China

**Keywords:** Giant cell angioblastoma, Bone tumor, Rare tumor, Pediatric malignancies, Interferon-α

## Abstract

Giant cell angioblastoma (GCAB) is an extremely rare soft tissue tumor of early childhood and only five cases have been described to date. As such the clinical, pathological, and prognostic features are poorly defined. We prensent here a new case of GCAB in bone of a child aged 4-years old. The lesion was composed of dense and loose cell regions. The dense regions were characterized by nodular, linear, and plexiform aggregates of oval- to spindle-shaped tumor cells around small vascular channels and interspersed with large mononuclear cells and multinucleate giant cells. The loose cell areas were characterized by distributed fibroblasts and abundant myxoid matrix, which diminished with patient age. Infiltrative growth was observed in some areas. Oval-to-spindle cells showed positivity for Vimentin, CD31 and CD34 staining, and partial positivity for smooth muscle actin. Mononuclear cells and multinucleate giant cells showed Vimentin and CD68 positivity. Seventeen months after thorough curettage of the lesion, a local recurrence was found. Based upon the clinical, histological and immunohistochemical findings, infiltrate condition, and prognosis, we classified GCAB into two subtypes. Type I does not infiltrate surrounding tissues and has good prognosis. Type II infiltrates the surrounding tissues, relapses earlier, and has worse prognosis. This report augments the limited GCAB literature to promote our understanding and guide therapy of this rare disease.

**Virtual slides:**

The virtual slide(s) for this article can be found here: http://www.diagnosticpathology.diagnomx.eu/vs/6699811297488137

## Background

Giant cell angioblastoma (GCAB) is an extremely rare soft tissue tumor of children. Diagnosis can be made according to the unique morphological characteristics, which include nodular, linear, and plexiform aggregates of oval- to spindle-shaped tumor cells interspersed with large mononuclear cells and multinucleate giant cells [1]. Since the first case of GCAB reported in 1991 [1], only one report described the other three cases of GCAB in infants occurring in the central palate, hypothenar portion of the right hand, and on the scalp, respectively [2]. We had reported the fifth case in the world of GCAB [3]. The involved tissues of GCAB in human pediatric patients have been reported as skin and soft tissue, including a substantial portion of an upper extremity [1], mucous membranes of the central palate, skin of the scalp, subcutaneous soft tissue of the right hypothenar eminence extending along the metacarpals [2], and leg bone [3]. Considering the extremely few case reports of GCAB worldwide, it is critical to include more case reports in the literature to increase our understanding of the clinicopathological features of GCAB. Here, we present the sixth GCAB case, which is quite different from the other five cases and occurred in the bone at the early childhood.

Iinterferon-alpha (IFN-α) has been successfully used as an antitumor therapeutic agent [4] and has shown robust antiangiogenic properties [5]. IFN-α has been used to treat two GCAB patients and showed remarkable wound healing properties after operative therapy, even promoting regeneration of bone [6].

## Case presentation

**Figure 1:**
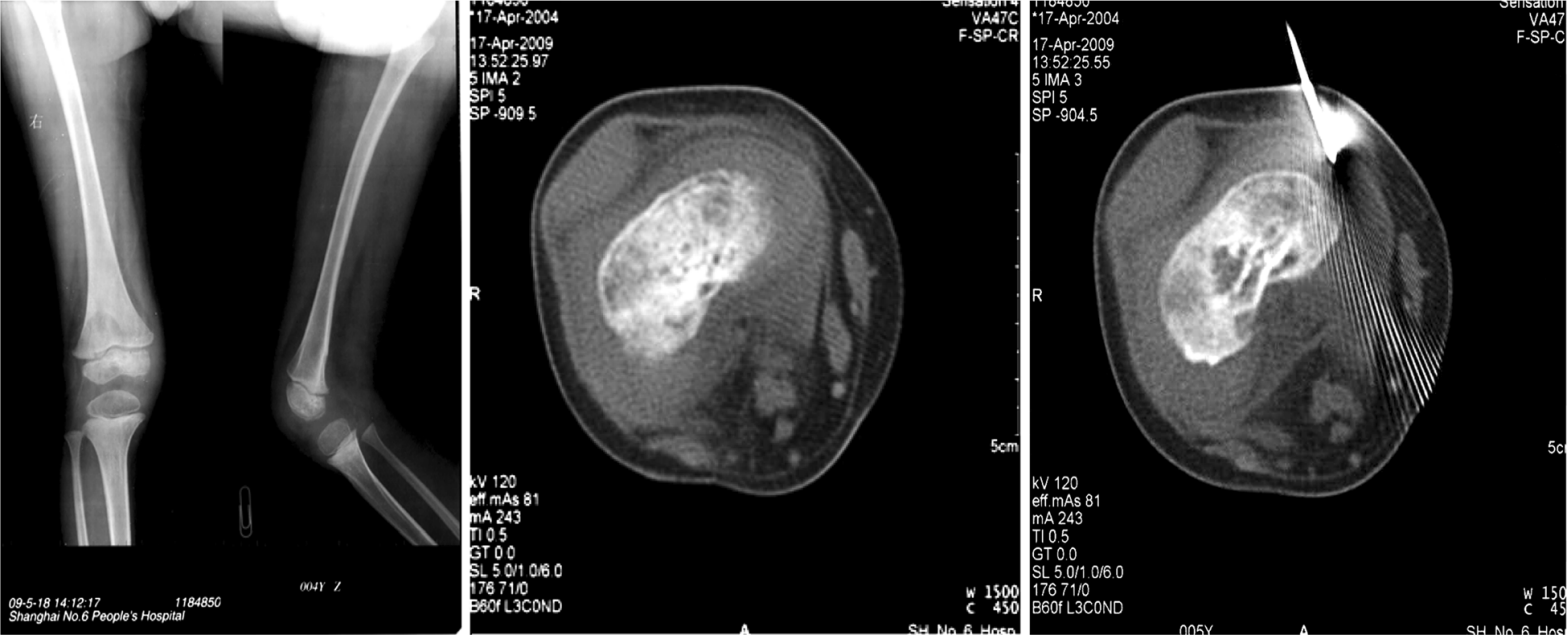
**X-ray and CT imaging of the right leg of the case.** A. X-ray showed that the pattern of bone was generally normal and there was no swelling of surrounding soft tissue. Density of the metaphysis of the lower right thighbone had irregularly reduced. B and C. CT scan showed the density of medullar of the lower right femoral metaphysis was asymmetrically reduced with irregular thickening of the cortex and swelling of soft tissue.

A 4-year-old boy, who was born from a full-term healthy pregnancy, was presented with the complaint of unsteady standing since one-year-old. The symptom was worsened gradually with increasing restricted mobility of the right knee joint and a slowly enlarging mass on the inner side of the right lower thighbone and surrounding soft tissues. Examination found a 3 × 3 cm^2^ relatively rigid mass with tenderness at the inner side of right knee. X-rays revealed that the medullary density of the right lower femoral metaphysis was irregularly reduced. CT imaging showed that the density of medullar of metaphysis was irregularly reduced and contained multiple small cysts. The cortex showed irregular thickening, and swelling was found in the proximal soft tissues. However, there was no obvious periosteum reaction (Figure 1). A thorough curettage of the lesion was performed. However, seventeen months later, MRI examination revealed a local recurrence in the operated area.

**Figure 2:**
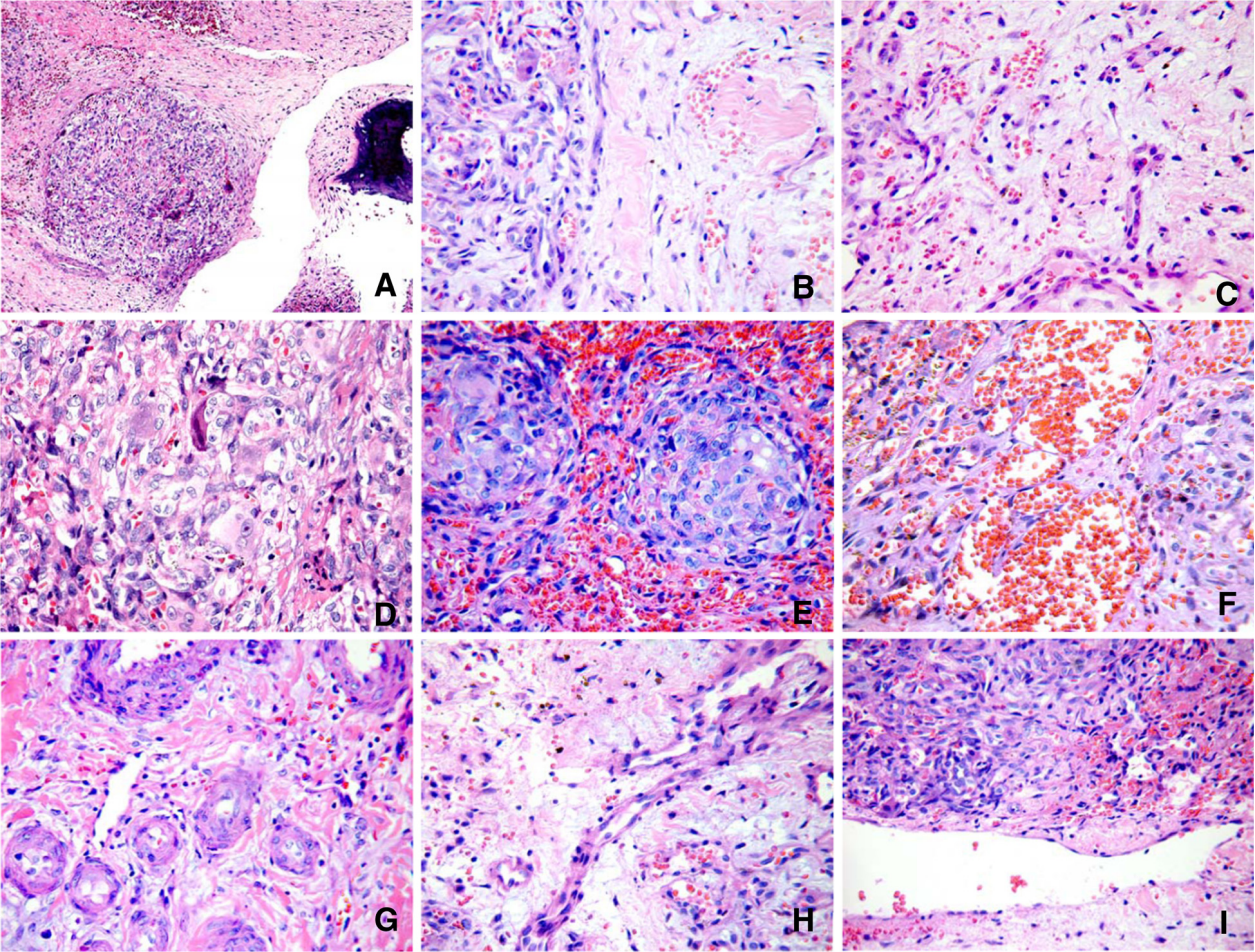
**Microscopic features of the case.A**. The tumor formed by dense and loose cell regions. The dense regions were composed of plexiform and nodular oval- to spindle-shaped tumor cells. The loose regions were composed of fibrosis. Bone trabeculae erosion was observed. **B**. There were relatively clear boundaries of dense and loose cell regions, both of which exhibited abundant capillary vessels. Muscle infiltration was confirmed by the few residual muscle cells in the tumor (arrow). **C**. The loose region was made up of two components. One was a fibrotic area with fibrinoid degeneration, and the other was composed of sparse stellate-shaped cells with myxoid background. **D**. The large mononucleate cells and multinucleate giant cells were randomly-distributed among the oval-to-spindle cells, with abundant capillary vessels in the mesenchyme. The osteoclast-like multinucleate giant cells were abundant and scattered randomly. **E**. The oval-to-spindle cells and giant cells aggregated to form non-necrotizing-like granulomas lesions, with abundant small vascular-formation. **F**. The sponginess hemangioma regions were also observed in the dense tumor cell nodules. **G**. The thick-wall vessels with periendothelial proliferation were congregated in some areas. This pattern always appeared in the loose region. **H**. The big vessels showed periendothelial proliferation and protruding into the lumen, as well. Stellate-shaped cells were sporadically distributed in the loose region. **I**. The big thin type of vessel was seen in some areas. (H&E stain. Magnification: A: 10×, B-I: 40×).

Microscopically, the tumor occupied the medulla and eroded parts of the cortex, but the remaining bone trabeculae still displayed prominent osteoblastic rimming. The first characteristic noted in the case was the distinctive nodular histological feature, which was composed of oval-to-spindle cells and formed abundant vascular networks. The oval-to-spindle cells had congregated and circled to form a central vessel-like structure, which was filled with red blood cells. These oval-to-spindle cells were considered likely to be the key of GCAB pathogenesis and had the ability to form a functional blood vessel within the tumor. The vascular endothelial cell markers-positive further confirmed that the oval-to-spindle tumor cells have the characteristics of vascular endothelial cells and pericytes. The second characteristic of GCAB was the abundance of mononuclear and multinucleate giant cells sprinkled throughout the tumor. The multinucleate giant cells and large mononucleate cells exhibited a distinctively strong expression pattern of CD68, which indicated a general histocytic lineage. The third characteristic of GCAB was the sparse presence of spindle- and spider-shaped cells, and mast cells, scattered throughout the loose mesenchyme of the tumor and accompanied by significant edema, myxoid or fibrosis in interstitial compartment. However, as this case was older than our former case (4-years *vs.* 15-months) and the history of tumor-associated symptoms was longer, this characteristic was not obvious. Therefore, it might suggest that the oval-to-spindle cells have a tendency to proliferate and replace the regions of loosened mesenchyme or collagen over the pathogenic course of GCAB. In addition, widespread deposition of hemosiderins was observed and might be included as the fourth characteristic of GCAB (Figure 2).

**Figure 3:**
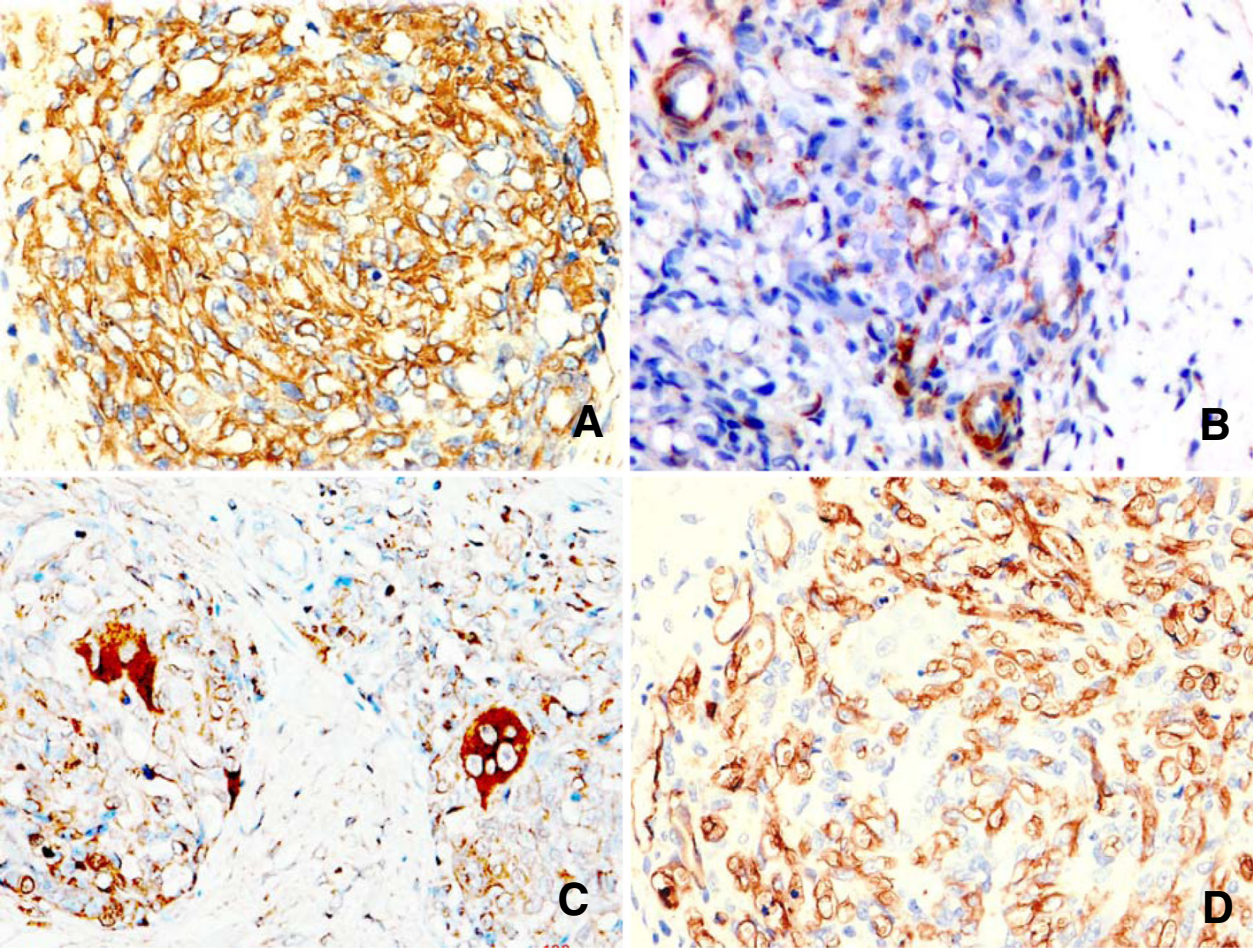
**Immunohistochemical staining of GCAB.A**. Vimentin-positivity throughout the tumor. **B**. SMA-positivity in perivascular cells of the well-developed vessels and basement membrane. **C**. CD68-positivity was strong in the giant cells, weak in some oval-to-spindle tumor cells. **D**. CD34-positivity in the oval-to-spindle tumor cells and the monolayered small channels, but not in the giant cells. (Magnification: A, B, and D: 40× C: 20×).

Immunohistochemical staining showed that most of the oval-to-spindle cells, large mononuclear cells, multinucleate giant cells, and periendothelial cells were strongly positive for vimentin, but uniformly negative for calponin, h-caldesmon, AE1/AE3, desmin, and CD1a. The perivascular cells of the vessels strongly expressed calponin and h-caldesmon. The monolayered small channels and oval-to-spindle cells in nodular or linear aggregate areas were positive for expression of vascular endothelial cell markers, CD31 and CD34, and partly expression of FVIII. SMA was most robustly expressed in the perivascular cells of the well-developed vessels and monolayered small channel in the hemangioma-like areas. Other regions of the tumor showed weak or no staining for SMA. The large mononuclear cells and multinucleate giant cells exhibited strong expression of the classic macrophage marker, CD68, but negative for Desmin, S-100, LCA, and SMA (Figure 3).

## Differential diagnosis and prognosis

Histological differential diagnosis mainly involves other vascular neoplasms accompanying giant cell proliferation, such as giant cell fibroblastoma [7], angiomatoid fibrous histiocytoma [8], plexiform fibrohistiocytic tumor [7,9]. The giant cells in Giant cell fibroblastoma are characteristically located in the inner-side of the cranny-like vasculature and CD34-positive, which is different from GCAB. Angiomatoid fibrous histiocytoma exhibits stretched tumor cells that are compartmentalized by cystic dilated vessel-like lumen, and have a compact envelope surrounded by abundant lymphocytes. Plexiform fibrohistiocytic tumors also form multiple nodulars, but lack vasculature and do not conform to hemangioma structures. The proliferated cells in plexiform fibrohistiocytic tumors are mononuclear histiocytes and fibroblasts. The osteoclast-like giant cells that sparked in the tumor lack clear, large nucleoli.

Intravascular lesions, such as reactive neoplastic lesions, with organized thrombus accompanied by central foreign material and an associated foreign body giant cell reaction should be carefully considered during diagnosis, and patient age and case history may help to distinguish the underlying disease [10]. In addition, as our case was derived from the tibia, it was important to rule out adamantinoma. Adamantinoma is characterized by biphasic epithelial and osteofibrous components and rarely occurs in children (median age at diagnosis: 25 to 35). Both of these features may be used to differentiate adamantinoma from GCAB [11]. Some of the malignant mesenchymomas, including leiomyosarcoma, rhabdomyosarcoma, chondrosarcoma, osteosarcoma and myxomatous components, should also be considered as differential diagnoses [12], and are distinguishable from GCAB by its lack of malignancy.

The prognosis of GCAB is not yet defined. Our follow-up to date has suggested that the prognosis of GCAB was relatively good but varied with site, patient age and the adjacent tissue infiltration. Tumor penetration of bone and infiltration of soft tissues might indicate early recurrence. And adjacent tissue infiltration would likely be the most significant marker for poor prognosis. Interferon-α therapy has been suggested as a preventative therapy against GCAB recurrence [2,6]. Neither of our two cases received IFN-α therapy, and the first case has shown no signs of recurrence within the three years of follow-up [3]. But this case recurred seventeen months after *en bloc* resection. Comparing the infiltrate condition of our two cases with all the other four reported cases led us to conclude that GCAB could be divided into two subtypes. Type I, as exemplified by our first case, occurs earlier, does not infiltrate surrounding tissues, and has good prognosis. Type II, as exemplified by the current case, occurs later and infiltrates the surrounding tissues, and is expected to relapse earlier and have worse prognosis. Type I could transform into Type II over long-term development. And IFN-α therapy may benefit for type II of GCAB.

## Conclusion

Our newly identified case of GCAB supports that GCAB as a distinct and rare angiogenic tumor in infants with local infiltration potential. We summarized the six collective GCAB cases reported to date and distinguished them into two subtypes. Diagnosing and distinguishing the subtypes of GCAB is meaningful for estimating prognosis and guiding therapy. Infiltration status is likely a key prognostic indicator of GCAB. A definite diagnosis followed by complete excision might facilitate better prognosis of GCAB. Interferon-α therapy benefits type II GCAB patients.

## Consent

Written informed consent was obtained from the patient for publication of this Case Report and any accompanying images. A copy of the written consent is available for review by the Editor-in-Chief of this journal.

## Competing interests

The authors declare that they have no competing interests.

## Authors’ contributions

MRJ collected the patient’s clinical information, JZM and ZHZ provided the case and were in charge of the patient, ZXZ revised the article. ZQL wrote this manuscript. All authors read and approved the final manuscript.
